# Is there a Future for Remote Ischemic Conditioning in Acute Myocardial Infarction?

**DOI:** 10.1007/s10557-020-07074-x

**Published:** 2021-03-05

**Authors:** Thomas Stiermaier, Yochai Birnbaum, Ingo Eitel

**Affiliations:** 1 University Heart Center Lübeck, Medical Clinic II, University Hospital Schleswig-Holstein, Ratzeburger Allee 160, Lübeck, 23538 Germany; 2 German Center for Cardiovascular Research (DZHK), Partner site Hamburg/Kiel/Lübeck, Lübeck, Germany; 3 The Department of Medicine, The Section of Cardiology, Baylor College of Medicine, Houston, TX USA

Immediate reperfusion of the infarct-related vessel by primary percutaneous coronary intervention (PCI) is the cornerstone of treatment for patients with ST-elevation myocardial infarction (STEMI) to limit myocardial injury [1, 2]. Several studies prove a decline in mortality following STEMI during the last decades in parallel with a greater use of primary PCI, modern antithrombotic therapy, and improved secondary prevention. However, mortality rates are still substantial, and the number of patients with post-infarction heart failure is on the rise [3]. Consequently, there is an inevitable need for additional treatment options to reduce post-infarction myocardial and microvascular damage and prevent adverse left ventricular remodeling and heart failure.

Remote ischemic conditioning (RIC) is one of the most innovative and promising approaches in this regard. Brief cycles of transient ischemia and reperfusion applied to an organ or tissue remote from the heart resulted in reduced myocardial damage in experimental models of acute myocardial infarction [4, 5]. In the clinical setting, the RIC stimuli can be simply delivered by the repetitive inflation and deflation of an upper arm blood pressure cuff, which makes RIC a non-invasive, low-cost adjunct to the established treatment options. However, the powerful cardioprotective effects of RIC in animal studies did not convincingly translate into the expected improvement in clinical outcome. Initial proof-of-concept studies in patients with ST-elevation myocardial infarction (STEMI) were promising with a significant reduction of myocardial injury assessed by biomarker release or cardiac imaging and a reduction of adverse events, mainly post-infarction heart failure, in smaller clinical studies and meta-analyses [4]. In accordance, the randomized RIC-STEMI trial (*n* = 448 patients) reported reduced rates of cardiac death and hospitalization for heart failure after additional RIC [6], and the long-term results of the LIPSIA CONDITIONING trial (*n* = 696 STEMI patients) also indicate a prevention of post-infarction heart failure after RIC in combination with ischemic post-conditioning (via repetitive brief interruptions of coronary blood flow immediately after reperfusion) [7, 8]. However, most recently the large international, multicenter, randomized controlled CONDI-2/ERIC-PPCI trial failed to show any beneficial effect of RIC in STEMI patients treated with primary PCI (*n* = 5401) on clinical outcomes (cardiac death, heart failure rehospitalization) [9]. Differences in the RIC algorithms (e.g., number and duration of limb ischemia/reperfusion cycles or RIC of arm versus leg) and the use of RIC alone rather than multi-targeted approaches such as a combination of RIC with ischemic post-conditioning are among the potential reasons for the failure to translate cardioprotective effects of RIC into superior clinical outcome. Furthermore, animal models of ischemia/reperfusion do not exactly resemble STEMI in a patient and comorbidities (e.g., hypertension or diabetes) and infarct characteristics (e.g., duration of ischemia or extent of ischemic myocardium) might impact the cardioprotective effect of RIC. In addition, adverse interaction with prescribed medications (e.g., aspirin) may dampen or mask the effects of RIC in the clinical setting [10, 11].

In this issue of the journal, Yu Zheng and colleagues present the rationale and design of the intelligent “Internet-Plus”-based full disease cycle remote ischemic conditioning (i-RIC) trial [12]. This clinical trial will randomize 4700 STEMI patients undergoing primary PCI at five hospitals in China to pre-, per-, and post-operative RIC combined with long-term i-RIC after infarction or to conventional treatment. The primary study endpoint is the combined 12-month rate of cardiac death and hospitalization for heart failure. The i-RIC trial is well designed and investigates several novel approaches in the field of RIC. The completely non-invasive conditioning protocol covers the whole disease cycle before, during, and for several weeks after primary PCI. Of note, the terms “pre“, “per,” and “post” in the present study protocol refer to reperfusion of the culprit vessel by primary PCI rather than the beginning of ischemia, which is the predominant reference particularly in experimental studies. Previous large clinical studies used either RIC before primary PCI (CONDI-2/ERIC-PPCI) [9] or post-conditioning by repeated balloon occlusions immediately after reperfusion of the infarct-related coronary artery and before stent implantation (DANAMI-3-iPOST) [13]. Both concepts failed to improve clinical outcome in patients with STEMI [9, 13]. The long-term results of the LIPSIA CONDITIONING trial, however, suggest that the combination of RIC and ischemic post-conditioning may improve clinical outcome by a reduction of heart failure events [8]. Therefore, an extended conditioning protocol covering the time of ischemia, reperfusion, and post-infarction myocardial healing and repair has the potential for additive cardioprotective effects and subsequently an improved clinical outcome. Another innovative aspect of the i-RIC trial is the fully non-invasive conditioning algorithm with an automated cuff inflation/deflation device and real-time monitoring of treatment adherence with a smartphone application during follow-up [12]. In contrast to previous studies, which used manual inflation/deflation of a blood pressure cuff, this approach allows a standardized, operator-independent application of the RIC stimuli. A relevant drawback in the study protocol is the use of clopidogrel rather than prasugrel or ticagrelor, which are the preferred P2Y_12_ inhibitors in patients with STEMI [1]. Furthermore, an extended follow-up beyond 12 months after infarction might be important since the protective effects regarding heart failure prevention, for example, in LIPSIA CONDITIONING, were observed on the long run several years after the index event [8]. However, the i-RIC trial will definitely add to our knowledge regarding the cardioprotective impact of RIC on structural/functional myocardial damage and clinical outcome following STEMI. Besides, the large study population potentially allows the identification of high-risk subgroups with particular benefits after cardioprotective approaches in addition to state-of-the-art reperfusion and medical treatment.

In conclusion, despite sobering results in the most recent RIC studies, RIC should not yet be abandoned and deserves further clinical evaluation. The i-RIC trial pursues some innovative approaches and will provide novel insights into the clinical value of RIC in patients with STEMI. In addition, experimental studies are required to elucidate the underlying mechanism and signal pathways of RIC in order to improve conditioning protocols and patient selection.
